# Future-making through eventing human-machine listening

**DOI:** 10.12688/openreseurope.17390.1

**Published:** 2024-04-24

**Authors:** Mona Hedayati

**Affiliations:** 1 Centre for Interdisciplinary Studies in Society and Culture, Concordia University, Montreal, Québec, H3G 1M8, Canada; 2 Antwerp Research Institute for the Arts, Universiteit Antwerpen, Antwerp, Flanders, 2000, Belgium

**Keywords:** Acoustic Experience, Human-Machine Symbiosis, Machine Listening, Future-Making, Affective Attunement

## Abstract

*Reverb-Resonate: Sounding the Affective Frequencies of Migration* operates at the intersection of art, science, and technology to articulate an emotional landscape of migration and exile. Rooted in the methodology of research-creation (RC) and grounded in the interdisciplinary field of Art, Science, and Technology Studies (ASTS), the project transcends conventional disciplinary boundaries to offer speculative possibilities through human-machine listening. Drawing on the body is an already augmented site, the project makes audible physiological sensors that capture micro-level intricacies responsible for stress regulation. Listening, is, thus, foregrounded as the core public engagement strategy, creating a layered sound collage that interweaves somatic registers of recorded breathing samples and physiological sensor values with machine listening to recreate new forms of sound. Engagement with ASTS is, hence, in the form of a method that traverses transforming sensor application, generating technicized sound and composing an acoustic experience capable of affective engagement. Through machine learning—a subfield of artificial intelligence—the notion of ‘machine listening’ extends beyond human hearing limitations, introducing non-normative structures to challenge and expand habitual forms of human listening. *Reverb-Resonate*, hence, leverages artistic strategies and techno-augmentations to address the crisis of imagination that hinders opening up to realities far from the familiar and the personal to imagine ‘what could be’ as a way of future-making. It underscores the critical edge of RC and ASTS in addressing complex critical issues, proposing a speculative space where the human-machine hybrid puts forth a socio-technical assemblage of listening to understand 'otherly' experiences. The project, thus, advances a critical inquiry into the mediation and augmentation of listening to imagine new possibilities for embodied engagement with unfamiliar emotional spaces and experiences.

## Context: From research-creation to art, science and technology studies

The following is a survey of theory and practice-based observations across the experiments I have and will conduct that fit within two rather peculiar orientations to scholarly work that stem from disrupting disciplinary boundaries. The grounding orientation is the one rooted in research-creation (RC) as a Canadian academic methodology that I situate my scholarly and artistic work within. As a close counterpart of the European framework of artistic research, RC does not separate research from practice but acknowledges that one necessarily informs the other as a way of making and disseminating new forms of knowledge. While as the name implies, RC maintains research and creative practice in a constant dialogue with one another, it tends to have an interventionist approach to conventional modes of knowledge production considering its focus on process-based and experimental inquiry. ^1^

RC, thus, carries a dual impact as it has the capacity to analyze and comment on—in the tradition of social sciences and humanities—and to produce new forms of knowledge intertwined with the analysis and the critique using the generative methods of artistic practice in its wide definition. My alignment with this tradition is rooted in the speculative power it has to offer which is in line with fields that have a critical bent as “they speculate about different times/spaces from a particular time/space, and in so doing shape and co-compose what could be.” ^2^ What could be, concretely in the case of my project, is building possibilities of experiencing forms of emotional space emanating from lived experiences that are unfamiliar through mobilizing the hybrid space of art-science-technology.

The latter construct, thus, touches upon the second orientation: the conglomeration of technoscience—an already hybrid of science and technology—and artistic inquiry as established within the interdisciplinary field of Science and Technology Studies (STS) as Art, Science and Technology Studies (ASTS). ^3^ The recent developments in technoscience, particularly intelligent machine systems and their agentive quality have further complexified the technological infrastructure, giving rise to the inclusion of these artefacts and processes as part of socio-political life. ^4^ In this sense, as machines become an indispensable part of human life, hence the term *socio*-technical systems, they allow for new ways of interacting with the world, at times as augmentations, and at other as amputations. ^5^

Within ASTS, these augmentations and amputations can then be made tangible leveraging the practice-based nature of art which in conjunction with RC can engage with critical commentary. These hybrid spaces then reinsert the fact that “science has failed to produce anything like the public sphere of art to entertain the cultural and ethical arguments we need to have about our technoscience.” ^6^ The way, thus, RC and ASTS imbricate allows for a new approach for a public-facing engagement and concretizes what is often hidden behind thick academic walls. Above all, these two orientations allow for machines and non-machines (here not only humans and machines but also agentive object-processes emanating from convergence of the two) to come together to be initially viscerally felt and consecutively critically processed thanks to the experience-building and event-making capacity of artistic practice. As this machine-nonmachine ensemble reorients the way the world is perceived, the capacity of mutual action brings attention to dimensions that humans could not have seen and hence could not have perceived to open up a critical capacity for better understanding of the self and the other. It is at this intersection that my ongoing project operates within, and my writing emanates from to put forth experimental, process-based work—streams of thinking and bursts of making—rather than reporting the outcome and findings after the fact. The goal behind such a sketching is to showcase the heterogenous coupling of sites of knowledge underpinning machine-nonmachine framework and their potential to produce speculative possibilities.

## The project: From body to sound

My ongoing project entitled *Reverb-Resonate: Sounding the Affective Frequencies of Migration* that has gone through several iterations and experimentations as it continues to do so, focuses on leveraging sound to convey the affective and emotional tone of my lived experience as a migrant in exile to audiences. Framed as ineffable and hence notoriously difficult to convey, the project formulates migration and exile as embodied experience with the goal to collect corporeal-level information from my body to make these registers felt through acoustic experiences. I, hence, draw on augmentations of somatic phenomena using sensors fit to capture such information from the body that makes visible the micro-level intricacies in metabolic and biochemical processes involved in regulating stress. In this scenario, the technoscientific extension to the body allows for capture and augmentation of otherwise invisible bodily processes but not as an uncritical enhancement of the body in a transhumanist quest ^7^ for enhancing biological processes. ^8^ Instead, the project aims to open up the possibility to bring awareness to intricacies of affective response felt in the body, where complex experiences are felt, using the affective language of the sound. In doing so, listening is taken up as a sensory prompt as the biological signals are transformed into acoustic signals to take an audible form.

In this sense, as the sensor readings constantly reshape and reshuffle the acoustic qualities, a different level of physiological affective response, that of recorded breathing rhythms creates a foundation for listening. On a complementary level, sensor readings then sensitize the ears by shifting the qualities of the sound as their qualities change. The sound is, thus, composed as a 3-layered collage where direct recorded samples are interwoven with *machine listening* output—a concept within computer music to generate new forms of sound from already existing ones—along with sensor values that interlace these two levels. As the capacity—or rather agency—of machines to listen to and reproduce sound, machine listening in this case uses machine learning to create an expanded and non-normative form of listening. The goal behind complexifying the sound by foregrounding novel forms of sound objects is to create a new sensorial space where the unfamiliarity of my experience can be felt through the eccentric qualities of sound.

The project, thus, takes the contemporary body as an already augmented-amputated technoscientific site to activate an ASTS method that comprises transforming the application of sensor as a technoscientific object, generating technicized sound, and devising artistic strategies to compose an acoustic experience based on these augmentations to circle back to body. The attention to body on a larger scale is then meant to underscore the close coupling between bodily sensations, emotions and lived experiences. These dynamics then leverage creative production to comment on complexities of migration and exile, the body as a site for emotion regulation, and techno-augmentation and hence tap into the “hyphenation” of RC that allows the synthesis of research and creation. ^9^ However, beyond the frameworks that this project feels at home with—RC and ASTS—the framing of machine-nonmachine as a way to probe the technicized and the non-technicized under a rubric for exploration and assessment is especially apt. This orientation, thus, enables dealing with sound making sourced from the body that juxtaposes the unquestionable material nature of this type of sound, human operation to materialize and later consume it, with machine operation to augment the body and technicize the sound. To focus on one aspect of the project and engage with an analysis rooted in the frameworks discussed, what follows looks into the act of listening, its personal, social and machinic aspects and the way the project activates these dimensions to build transformative experiences.

## Sociology of listening: The personal, the social, the technical

Analyzing the three levels of listening—personal, collective, and machinic—that the artistic process incorporates can elucidate how listening is central to the project. The first question that emerges is what is listening in the first place? As Jonathan Sterne aptly points out, listening ought to be differentiated from hearing as it is a learned capacity as opposed to a biological function. Hence, there is a level of entrainment already built into the experience of listening in the form of techniques culturally learned to attune the ear to sounds. With modern-day technicization of listening then specific practices were developed to assign logic and objectivity to listening that Sterne refers to as “the Audile technique.” ^10^ Along the path of entrainment, different qualities of listening add layers of nuance into the formalized experience particularly evident in the two modes of *causal listening* and *reduced listening*. While causal listening intends to collect information about the source, reduced listening engages in exploring the sonic qualities and textures regardless of semantic qualities. ^11^ On a personal level and in the absence of linguistic communication and direct semantics, the listener, thus, still looks for cause-effect relationship as a historically conditioned reflex. ^12^ It is, thus, only in the process of loosening up causal listening with the intent to disrupt such patterns that the reduced listening come into play to open up the imagination.

Beyond the personal level, in the collective setting, listening becomes more of an interconnecting social experience when making personal-level deductions are challenged through reduced listening and attention is paid to qualities of sound and their impact on the body. However, collective listening does not require a radical transformation from personal to social to begin with as it seems to already have an inherent sociality built into it since the listening subject is already a social subject that socializes through the act of listening. In his book *Introduction to Music*, Theodor Adorono opens up by this provocation: “asked to say offhands what a sociology of music is, one would probably start by defining it as knowledge of the relation between music and the socially organised individuals who listen to it.” ^13^ Thus, a listening experience in a public context—whether music, sound art, broadcast, or speech—assumes a social position superimposed over the personal experience. The sociology of listening within the framework of this project, thus, pays attention to the networked experience of listening beyond the individuation of the sound within a complex sphere of socio-technical assemblages that encompass the personal, the social and the technological.

## Listening to machine listening: A speculative space

The latter structural element, the technological, touches upon the phenomenon of machine listening embedded in the history of technicization of listening, from techniques to apparatuses aimed at expanding the scope of listening. Within this domain, while numerous publications have addressed technical implementation of machine listening, in this context using machine learning for sound generation, ^14^ a dearth can be identified on the theoretical-critical level and how the phenomenon is figured into the human imaginary. I will, thus, touch upon machine listening as a strategy to get passed the human hearing limitation which concurrently then gets folded into vast socio-technical assemblages as an indispensable part of human listening practices. Machine listening, in this vein, addresses the deficit of human listening and sensemaking by way of recognizing machine to have a capacity “to listen more.” ^15^ While listening has classically been bracketed as a solo experience where subjectivity is the cornerstone of listening, the transformation of human listening through technological mediation has already challenged the strict subjectivity associated with listening as a complementary stance to the sociology of listening argument. As Domenico Napolitano and Renato Grieco assert, machine listening further challenges the paradigm of listening as it operates on its own logic far different than that of the human logic of listening, “insofar as it is the materialization of nonhuman and desubjectivized listening.” ^16^ By mobilizing the concept of the socio-technical—the one central to the STS epistemology highlighting the inseparability of the two phenomena—they then argue that the act of listening fundamentally occurs between the human and machine. This argument is not a simplification but an acknowledgement of the complexity of different layers and processes involved in technological listening that engages humans and their extended technological apparatuses which Napolitano and Grieco refer to as *the folded space*. ^17^ Thus, while machine listening holds fundamentally different qualities in relation to human listening, its output is meant to get folded into the human listening experience to form augmented listening as a distinct socio-technical imaginary.

This argument is in line with the concept of human-machine hybridities vastly discussed in the STS scholarship, ^18^ to reckon with the increased permeability of boundaries between humans and machines. More importantly these two distinct logics are negotiated in a mutual substrate to achieve a certain goal, highlighting a reciprocity that goes beyond machines or humans assuming a submissive role. In the particular case of art produced using machine learning, the level of autonomy that the machine assumes can be shockingly lively in comparison with classic artistic media as it appears “strange, surprising, indeterminate, unfathomable, uncanny, unexpected, unpredictable, unexplainable, and unknowable.” ^19^ In this sense, machine listening assumes an ‘animate’ quality where the phenomenon of listening more teases the human listening habits and the audible techniques sharpened historically. The folded space then becomes a space of a new audile technique to be developed where these novel qualities of listening become part of the human listening spectrum.

 Machine listening operating at the intersection of machine learning ^20^ and artistic practice then leverages the capacity of machines to tap into the already ubiquitous remix culture brought about by the legacy of computer art to recycle existing forms and create new recombinations as a way to speculate the future. ^21^ In this process the human and machine action are inseparably fused together to create a hybrid where both exert agency to reach a common goal. In this sense, as during the training process machines develop new behaviors in an adaptive manner over which the artist have only an indirect control, the autonomy of the machine means the desired result may only be achieved through a process of trial and error, ^22^ in order to achieve a common ground of mutual intelligibility. ^23^ In the context of machine listening, this degree of autonomy then allows for true improvisational tones to come through, creating forms that at times can be radically different from human-centered synthesis logic. As a result, different pathways of speculation can become activated through phases of reduced listening that underlines formal qualities over familiar sonic maneuvers in the cause-effect chain of causal listening. The new space of audile techniques then goes beyond the crisis of imagination that hinders opening up to realities far from familiar and personal narratives to imagine *what could be*.

## Machine-nonmachine assemblage: Expanding senses

On a pragmatic level, to highlight the critical perspective of the project, machine listening is a strategy to tap into the speculative possibilities of listening as a way to bring awareness to emotional tones unfamiliar to the listener. As the recorded breathing rhythms create the material for the machine listening, the reproductions captured as the result illustrate what listening more can mean. From the perspective of habitual human-centered listening, the machine listening operation in this project can appear as operating on a warped scale as it confuses and conflates that which is close with that which reverberates at a distance, exaggerates the frequency and the repetition, and sharpens some textures while muffling others. The slightly awkward quality of these reproduced sounds that make sense while they do not then bring further attention to the experience of listening that falls between causal and reduced listening. The project, hence, puts forth a further expanded acoustic collage that integrates the machine listening result along with my selection of some of the original recorded rhythms of breath to underscore the differences in sonic qualities and sharpen the sense perception.

In this vein, the machine-nonmachine assemblage involves multiple actors: human (and its micro and macro materiality) and machine (as hardware and software—computer, sensor, and their circuitry, software and code including algorithms) along with their products as biological and acoustic signals. This assemblage can then make felt the affective intricacies that take up a shape, that of listening, as an embodied experience. This level of feeding the somatic intricacies—biological signals from sensors and breathing rhythms—back into the body for a transformative emotional impact through human sense perception then makes use of the technoscientific, the artistic, and the critical to create a space for embodied understanding of unfamiliar lived experiences. This new assemblage of listening that occurs between the machine and nonmachine foregrounds audition to tap into the future-making qualities of technicized sound reproduction, laying down experiences and possibilities beyond the quotidian firsthand personal experience. Future-making in this sense, goes beyond the linear temporal construct of past, present, and future to instead tap into *futures* as multiplicity of possibilities taken shape as “imaginary ‘others’ outside the ordinary” to open up towards alternative pathways. ^24^

As the project iteratively takes different shapes through experimentations, it makes use of already-established frameworks discussed to design various acoustic collages and adjust the codes of interaction with audience in different performative settings. The goal is then to rehearse different ways of future-making to transduce affective resonances tied to my experience towards creating the conditions for affection, i.e., the quality of being attuned to an affective and emotional space, that of mine. As the project posits technicized listening as an active site for expanding sensory capacities, it imagines the possibility to tap into unknown emotional spaces and by extension unfamiliar experiences. In this vein, the project becomes a critical inquiry into the ways machines can mediate and augment our capacity for understanding ‘otherly’ experiences by expanding the universalized and habitual forms of navigating the world. It suggests that through the creative application of machine listening and integration of biological and acoustic signals, new pathways for comprehending the nuanced intricacies of experiences that might otherwise remain inaccessible can be opened. In this manner, *Curves & Reverbs* remains faithful to the urge for experimentation, the drive for subversion and heterogenous recombination as the crux of practices that grapple with the uncomfortable task of conducting research under the rubric of art-science-technology to highlight critical and problem-oriented perspectives.

## Ethics and consent

Ethical approval and consent were not required.

## Data Availability

No data are associated with this article.

