# Supplementary material for: Future-making through eventing human-machine listening
Source: Open Res Eur. 2024 Apr 24;4:79. [Version 1] doi: 10.12688/openreseurope.17390.1 (PMC11208846; doi:10.12688/openreseurope.17390.1)
Supplement: Peer review history

# Reviewer report: Reviewer response for version 1

**DOI:** 10.21956/openreseurope.18795.r41400

**Published:** 2024-07-22

**Authors:** Alexander Refsum Jensenius

**Affiliations:** 1 University of Oslo, Oslo, Norway

This exciting manuscript describes the ongoing research-creation project "Reverb-Resonate: Sounding the Affective Frequencies of Migration." I found the opening contextualization and reflection helpful and the theoretical discussion interesting. One challenge is that the article aims to incorporate many terms from different disciplines, which makes it somewhat challenging to follow some of the arguments (see details below).

 My most significant criticism relates to the article's lack of a description of the actual research project it discusses. After reading the article, I still do not understand much about what the research-creation process has entailed or the artistic, experiential, and exploratory products it has produced. What types of sensing have been used? How has the data been sonified? What types of machine learning have been employed? Have others than the author been involved in the process? Where have the results been displayed?

 In sum, while I am highly sympathetic to the topic and approach, I would request the following changes to be made to accept the manuscript:

1. Clarify (and perhaps simplify) some of the terminology used and present it in a way that makes it welcoming to read for researchers and practitioners from neighboring (inter)disciplines.
2. Present (at least briefly) the actual project so that the theoretical discussion is understandable.

Here are some more specific comments:

- I find the abstract too long for such a short article. I suggest shortening it slightly and presenting it in only one paragraph.
- Can you explain what you mean by "disrupting disciplinary boundaries"?
- It may be that "research-creation (RC) as a Canadian academic methodology". However, this approach is also outside of Canada, so writing this piece in a larger international context would be better.  
- I do not understand what you mean by "nonmachines (here not only humans and machines but also agentive object-processes emanating from convergence of the two)". I would think that your pairing of "machine-nonmachine" describes the convergence between the two. But what, then, is the nonmachine?
- Word pairs like "machine-nonmachine" should use an en-dash (instead of a hyphen) to denote the connection.
- As far as I can see, physiological sensors have been used, but no details are given. If so, I don't think you actually measure "metabolic and biochemical processes", so this should be corrected or explained.
- I don't understand the sentence "listening is taken up as a sensory prompt as the biological signals are transformed into acoustic signals to take an audible form." What is a sensory prompt? I guess you mean perception? How are tbe biological signals transformed?
- What do you mean by "contemporary body as an already augmented-amputated technoscientific site". What is a "contemporary body," and how does it differ from other (non-contemporary) bodies? What is a "technoscientific site"?
- I like the term "technicized sound", although I have never heard it before. I guess it is more common to use "mediated sound", but is that different from what you mean?
- How do you define "entrainment"? I think of it as the process leading to synchronization (typically to rhythms), but how does that work when you write "there is a level of entrainment already built into the experience of listening"?
- What do you mean by "socio-technical assemblage"?

Is the argument persuasive and supported by appropriate evidence?

Partly

Is the work clearly and cogently presented?

Partly

Is the topic of the essay discussed accurately in the context of the current literature?

Yes

Does the essay contribute to the cultural, historical, social understanding of the field?

Yes

Reviewer Expertise:

Music technology, sound and music computing, embodied music cognition

I confirm that I have read this submission and believe that I have an appropriate level of expertise to confirm that it is of an acceptable scientific standard, however I have significant reservations, as outlined above.

# Reviewer report: Reviewer response for version 1

**DOI:** 10.21956/openreseurope.18795.r41396

**Published:** 2024-07-13

**Authors:** Michele Geronazzo, Andrea Gulli

**Affiliations:** 1 Department of Engineering and Management, University of Padova, Vicenza, Italy; 2 Department of Mathematics, Computer Science and Physics, University of Udine, Udine, Italy

Mona Hedayati's essay "Future-making through eventing human-machine listening" proposes a theoretical framework at the intersection of art, science, and technology studies (ASTS). It treats the integration of human and machine listening as a possible vehicle to deliver affective attunement regarding the migration topic, creating possible future interactions and shared sensitivities via the Research-Creation (RC) methodology. 

 The author takes as an illustrative implementation the ongoing artistic (?) project entitled “Reverb-Resonate: Sounding the Affective Frequencies of Migration.” The work presented is at the intersection of different theoretical schools of thought: McLuhan's media studies, Adorno's studies on the sociology of music, and Donna Haraway's more recent works on cyborgs that criticize anthropocentrism. This background provides a solid theoretical framework for the project's intent of possible future social interactions and affective engagement through listening.

 Although such interdisciplinary studies are very welcome among academic works, and such cross-knowledge can inspire different scientific fields, the essay seems to be out of focus and confusing for three main reasons.

1. First, the project referred to is never clearly explained, nor is a reference given to allow the reader to gather more information about it.
2. The concept of machine listening is never explained in depth; the opportunities and capabilities attributed to it by the author could be the same as those of the more traditional digital signal processing techniques, and no elements are provided to distinguish between the two. Machine listening can easily be confused with the computer science discipline dedicated to modeling the action of listening and its fundamental components, e.g., sound source identification, localization, and diarisation.
3. Some theoretical concepts need clarification. In several sections, a listener's personal experience is considered as historically acquired knowledge and belief, e.g., the evolutionary concept of reduced listening, the biased identification of machine listening with machine learning approaches, the framework of embodied mind and sound-based practice for stress reduction, to name but a few.

 It is fundamental for the scientific validity of this essay to take care of the three aspects highlighted, in particular the technical aspects of "Reverb-Resonance: probing the affective frequencies of migration": which sensors the author employed, which automatic listening techniques the author used, which results led to the hypotheses of the essay.

 Furthermore, some ideas are taken for granted, without references, explanations, or results to support them: the crisis of imagination, the link between the remix culture and computer art (the conceptual relocation of an object dates back at least to Duchamp’s ready-made), the idea that biofeedback can lead to an affective and emotional relationship. What is the relationship with the scientific literature in the field of research through design (RtD)?

 Finally, the language must be revised; the sentences are too long, some terms need to be replaced with synonyms, and there are typos (“Adorono”). 

 Does non-machine assemblage mean the involvement of animals or plants?

 Some suggestions for revision:

- A reduction of the introduction, disambiguating personal ideas from established schools of thought.
- The inclusion of a section dedicated to the project “Reverb-Resonate: Sounding the Affective Frequencies of Migration” (or at least a figure explaining its workflow), and another for the topic of migration, which is completely overlooked.
- A stronger focus on the sociological aspects of listening related to this specific subject.

Is the argument persuasive and supported by appropriate evidence?

No

Is the work clearly and cogently presented?

No

Is the topic of the essay discussed accurately in the context of the current literature?

Partly

Does the essay contribute to the cultural, historical, social understanding of the field?

Partly

Reviewer Expertise:

Human-computer interaction, sonic interaction design, spatial hearing and computational acoustics

We confirm that we have read this submission and believe that we have an appropriate level of expertise to confirm that it is of an acceptable scientific standard, however we have significant reservations, as outlined above.

# Response

**Published:** 2024-07-14

**Authors:** Mona Hedayati

**Affiliations:** CISSC, Concordia University, Montreal, Québec, Canada

I would like to thank Michele Geronazzo and Andrea Gulli for taking time to write a comprehensive review on my contribution. Please see my response to the points made, numbered according to reviewers’ remarks:  

 1-     The project was specifically mentioned to get at the concept of “machine listening” and the analysis behind it and not as a case study to be analyzed. But perhaps it is indeed a good idea to clearly describe this intention in my revision while further expanding on relevant aspects of the project to the concept of machine listening. Please note that there are different contribution types under Open Research Europe (ORE) framework and reviewers in general conflate these differences in their remarks, concretely between a research paper, a case study and an essay. The current contribution being a conference proceeding is supposed to reflect the content of the conference presentation and hence was suggested by ORE editors to either go under an essay or an open letter, article types radically different than those the reviewers tend to push this contribution towards. Please note that under arts and humanities, the field I have chosen to see the relevant article guidelines under, there are 9 different article types, essay being one of those.  

 2-     I discuss machine listening “as the capacity—or rather agency—of machines to listen to and reproduce sound, machine listening in this case uses machine learning to create an expanded and non-normative form of listening.” This definition clearly identifies that machine listening is intersected with machine learning in the essay. However, further down I clearly explain that the whole point to bring this intersection up is to construct an “imaginary” to go beyond human listening and in fact I do draw a spectrum purposefully. My point is not to distinguish between different ways the term can be interpreted technically, even though my citations clearly point to what I define. This is evident in the opening of the passage “Listening to Machines Listening”: “the latter structural element, the technological, touches upon the phenomenon of machine listening embedded in the history of technicization of listening, from techniques to apparatuses aimed at expanding the scope of listening. Within this domain, while numerous publications have addressed technical implementation of machine listening, in this context using machine learning for sound generation, a dearth can be identified on the theoretical-critical level and how the phenomenon is figured into the human imaginary. I will, thus, touch upon machine listening as a strategy to get passed the human hearing limitation which concurrently then gets folded into vast socio-technical assemblages as an indispensable part of human listening practices. Machine listening, in this vein, addresses the deficit of human listening and sensemaking by way of recognizing machine to have a capacity to listen more.”  

 3-     I am unsure of what the reviewers mean by “a listener's personal experience is considered as historically acquired knowledge and belief.” In any case, I believe that while reviewers have clearly identified this contribution to operate across disciplines, they still assume a strict disciplinary position, specifically from engineering and computer science, and in fact most comments demonstrate this point. This contribution is far from a “technical paper”: in fact, it is neither technical, nor a paper. This “essay” is classified under the collection “Non-Machines: Playground of Perspectives,” visibly marked above the contribution. A quick glance over the description of the collection will shine a light on the fact that the essay is the proceeding of an “artistic” conference and hence there need not be a “(?)” in opening of the review, of whether or not this is an artistic contribution. This is above and beyond the fact that I dedicated an entire section as the introduction to situate my work within the academic tradition of “research-creation” as well as “art, science, and technology studies” with relevant references. On the last remark regarding reviewers’ comment on the “scientific validity,” please see the above point on the positioning of this contribution, neither being a technical nor a scientific paper. This is an artistic contribution, classified as an “essay” vs. a “research paper” or a “case study” under the collection of an artistic conference and arts & humanities article type (not even natural or social sciences). The fact that I do borrow across disciplines does not position this contribution as an engineering or CS contribution and in fact there is no rational justification on why it has to be treated as a scientific contribution, other than the fact that the disciplinary lineage of the reviewers dictates so.  Regarding why there is no mention of “research through design,” I underline the fact that I situated this essay as a contribution to “research-creation” and “art, science, and technology studies” and not “research through design” and I wrote an entire section on the relevance of these lineages. On the question of supporting arguments, I do not see the reason why there is a necessity to go back all the way to Duchamp here to explain the concept of remix culture while I do cite Manovich’s scholarship. I have not committed myself to drawing a comprehensive genealogy of the term or a lit review on the topic anywhere in the contribution.   On the remark that “the language must be revised,” while I understand that within the disciplinary value system of the reviewers the use of “must” might be acceptable, it is indeed less so in arts and humanities, the field that I have chosen and committed to its guidelines as outlined by ORE. While, in order to solve a particular engineering problem there “must” be certain protocols that need to be followed, in the case of arts and humanities, using “must” can come across as jarring, particularly in a review scenario. Moreover, while this essay does operate across disciplines, its contribution remains in the disciplinary tradition of arts and humanities within which long sentences are not uncommon. I cannot, however, be sure what the reviewers intend by “some terms need to be replaced with synonyms.”   Final points: thank you indeed for pointing out the misspelling of “Adorno.” To respond to “does non-machine assemblage mean the involvement of animals or plants?” I would encourage the reviewers to read the collection description “Non-Machines: Playground of Perspectives” to grasp how the conference organizers define the term and also to refer to my description where I refer to the term as “machines and non-machines (here not only humans and machines but also agentive object-processes emanating from convergence of the two).”   Finally, I would like to sincerely thank both reviewers again for taking time to write a detailed review, while recentering the review criteria for essays in arts and humanities: “essays are analytic or interpretive compositions on a single topic. This could include articles outlining an argument or personal point of view. The peer review focuses on whether the research methods used are appropriate, and the claims in the paper are sound and have academic merit, not on the perceived impact of the work. Open Research Europe encourages any reflective arguments and personal points of view in the arts and humanities.” While this contribution has already been approved by two reviewers, I continue to meticulously study and eagerly learn from every single review, drawing from relevant and constructive points made to revise this contribution.

# Reviewer report: Reviewer response for version 1

**DOI:** 10.21956/openreseurope.18795.r40992

**Published:** 2024-06-26

**Authors:** Eunseob Kim

**Affiliations:** 1 Purdue University, West Lafayette, Indiana, USA

The essay "Future-making through eventing human-machine listening" by Mona Hedayati explores the integration of human and machine listening to create speculative futures, particularly focusing on the affective frequencies of migration. The project, "Reverb-Resonate," leverages the methodology of Research-Creation (RC) within the interdisciplinary framework of Art, Science, and Technology Studies (ASTS). It utilizes physiological sensors to capture stress-related data from the body, transforming these biological signals into acoustic experiences. By foregrounding listening as a public engagement strategy, the project aims to create a layered sound collage that interweaves somatic registers with machine-generated sound. This hybrid approach seeks to address the limitations of human listening, introduce non-normative listening structures, and imagine new possibilities for understanding unfamiliar emotional experiences.

 The essay is well-situated within the context of current literature, drawing on recent developments in ASTS and RC. The author references a wide range of sources, including foundational texts in technoscience, human-machine symbiosis, and the socio-technical dimensions of listening. This grounding provides a solid theoretical framework for the project's exploration of speculative futures and affective engagement through sound.

 While the essay presents an innovative and compelling exploration of human-machine listening, its clarity can be improved. The abstract, in particular, is dense and challenging to penetrate. Simplifying the language and breaking down complex concepts into more digestible parts would greatly enhance readability. Additionally, more structured explanations and clearer transitions between sections would help the reader follow the author's line of reasoning more easily.

 My suggestions are below:

 1. Simplify the abstract to clearly convey the main objectives, methods, and findings of the project. Avoid overly technical jargon and ensure the abstract provides a concise summary of the essay.

 2. Use subheadings to organize the content better and provide clear transitions between sections. This will help guide the reader through the essay's arguments and evidence.

 3. Provide more detailed explanations of key concepts, such as "machine listening" and "affective frequencies," to ensure that readers from diverse backgrounds can understand the discussion.

 The argument is intriguing and supported by relevant literature, but certain aspects require further development to be fully persuasive. While the essay effectively draws on existing research to support its claims, it would benefit from more concrete examples and detailed descriptions of the project's implementation and outcomes.

 My suggestions are below:

 1. Include specific examples of how the physiological sensors capture stress-related data and how this data is transformed into acoustic experiences. This will help illustrate the project's process and impact more clearly.

 2. Elaborate on the results of the project's experimental iterations. Discuss any observed changes in audience perception or engagement with the sound collages.

 3. Where possible, include empirical data or case studies that demonstrate the effectiveness of the proposed human-machine listening approach in achieving its stated goals.

 The essay makes a significant contribution to the cultural, historical, and social understanding of the field by exploring the intersection of art, science, and technology in addressing complex emotional and social issues. The project's focus on migration and exile through the lens of human-machine listening offers a novel perspective that enriches the discourse on socio-technical assemblages and the role of artistic practices in speculative future-making.

 The essay presents an innovative and interdisciplinary approach to understanding migration and exile through human-machine listening. While the presentation and clarity can be improved, the work's theoretical grounding and potential contributions to the field are significant. With revisions to enhance readability and provide more concrete evidence, this essay will be a valuable addition to the literature.

 Additional comments are below.

 1. Although the author states that ethical approval and consent were not required, it would be helpful to briefly discuss any ethical considerations related to the use of physiological sensors and the transformation of personal data into public acoustic experiences.

 2. Consider including a section on potential future directions for the project, such as expanding the scope to include diverse populations or integrating additional sensory modalities.

 Overall, this essay is a commendable effort that bridges multiple disciplines to explore important socio-technical issues. With the suggested revisions, it has the potential to make a substantial impact on the field.

Is the argument persuasive and supported by appropriate evidence?

Partly

Is the work clearly and cogently presented?

Partly

Is the topic of the essay discussed accurately in the context of the current literature?

Yes

Does the essay contribute to the cultural, historical, social understanding of the field?

Yes

Reviewer Expertise:

Human-Machine Interaction, Acoustic Ecology, Sound Art and Sonic Practices

I confirm that I have read this submission and believe that I have an appropriate level of expertise to confirm that it is of an acceptable scientific standard.

# Reviewer report: Reviewer response for version 1

**DOI:** 10.21956/openreseurope.18795.r40988

**Published:** 2024-06-21

**Authors:** Eleni Ikoniadou

**Affiliations:** 1 Royal College of Art, London, England, UK

The essay draws on the artistic project Reverb-Resonate: Sounding the Affective Frequencies of Migration, which centers listening by making use of technical and scientific means to bring attention to the experience of migration and exile. Relying on recent literature in Art, Science and Technology studies (ASTS) and the practice-based method of Research-Creation, the author proposes new connections between human and non-human (machines and object-processes) ensembles, as activated by their artistic project. This essay offers an analysis of these possibilities, particularly through the act of listening, which is opened up to include machine listening in the age of AI. The argument is at its most convincing listening is posited as an activity that fundamentally occurs *between *human and machine as opposed to belonging to either. Such an approach moves the conversation from the trite oppositional debate on humans versus machines to the more complex “assemblage”, “hybrid”, “merging” relations or our socio-technical reality. This project and its analysis are part of a much-needed ongoing shift in literature and methodological and philosophical approach, whereby the speculative potential offered by practice-based experiments can bring awareness to the unfamiliar and unknown states of being and feeling of the ‘other’, away from the ignorant and limiting perspective of those who own the dominant narratives. As the author explains, “Future-making in this sense, goes beyond the linear temporal construct of past, present, and future to instead tap into *futures* as multiplicity of possibilities taken shape as “imaginary ‘others’ outside the ordinary” to open up towards alternative pathways.”

 My only suggestion would be to revisit the abstract, if possible, as it stands it’s a little difficult to penetrate and doesn’t do the more sufficient analysis that follows any justice.

Is the argument persuasive and supported by appropriate evidence?

Yes

Is the work clearly and cogently presented?

Yes

Is the topic of the essay discussed accurately in the context of the current literature?

Yes

Does the essay contribute to the cultural, historical, social understanding of the field?

Yes

Reviewer Expertise:

NA

I confirm that I have read this submission and believe that I have an appropriate level of expertise to confirm that it is of an acceptable scientific standard.
